# A Systematic Review and Meta-Analysis for the Association of Gene Polymorphisms in RAN with Cancer Risk

**DOI:** 10.1155/2020/9026707

**Published:** 2020-01-16

**Authors:** Yanke Li, Fuqiang Zhang, Chengzhong Xing

**Affiliations:** Department of Anorectal Surgery, The First Hospital of China Medical University, Shenyang, Liaoning 110001, China

## Abstract

As an important component of miRNA processing genes, RAN gene encodes the ras-related nuclear protein, which is a unique member of the Ras superfamily of GTPases. The mutations in RAN gene are very likely to play a critical role in pathology-related changes to miRNA transport and expression and thus participate in tumor genesis and development. Currently, accumulating studies have explored the association between RAN SNPs and cancer risk. However, the results are conflicting. In the present study, we performed a systematic review for the association of RAN SNPs with overall cancer risk. Meanwhile, a meta-analysis was conducted based on available data, aiming at clarifying the association between RAN SNPs and cancer susceptibility. After literature search and data extraction, 17 studies containing four RAN SNPs were involved in the systematic review. And 12 studies with two highly studied SNPs (RAN rs14035 C>T and rs3803012 A>G) were included in the final meta-analysis, consisting of 7662 cases and 9807 controls. The results showed that the rs14035 polymorphism was linked to a decreased cancer risk in overall subjects and hospital-based (HB) subgroup, while the rs3803012 polymorphism conferred to an increased cancer risk in overall subjects and population-based (PB) subgroup. Our findings suggested that the two SNPs had the potential to be predictive biomarkers for cancer risk. The study would provide novel clues for the identification of miRNA-related genetic biomarkers applied to predicting cancer susceptibility.

## 1. Introduction

As a group of endogenous small noncoding RNA molecules, microRNAs (miRNAs) have been implicated in a wide diversity of basic cellular function by downregulating the expression level of their target genes [1]. It has been well acknowledged that miRNAs are extensively involved in human cancer via regulation of various protooncogenes and tumor suppressor genes [2, 3]. They are generated in reliance on the precise coordination of miRNA processing machinery proteins. The global or specific deregulation of key genes in the miRNA biosynthesis pathway may influence regular production of mature miRNAs, thus leading to malignant transformation [4–6].

As an important component of miRNA processing genes, RAN gene encodes the ras-related nuclear protein, which is a unique member of the Ras superfamily of GTPases. It is an essential ingredient for the transportation of pre-miRNAs from the nucleus to the cytoplasm through the nuclear pore complex in a GTP-dependent manner [7, 8]. RAN is overexpressed in many cancer cell lines, including the stomach, colon, pancreas, lung, and ovarian cancer [9–11]. Moreover, RAN protein is also a well-known downstream modulator of the PI3K signaling pathway, which mediates cancer cell invasion and metastasis [12]. Therefore, the mutations in RAN gene are very likely to play a critical role in pathology-related changes to miRNA transport and expression and thus participate in tumor genesis and development.

Single nucleotide polymorphisms (SNPs) are the most common form of genetic variation in human genomes and also widely exist in the RAN gene. Currently, accumulating studies have explored the association between RAN SNPs and cancer risk. However, the results are conflicted. For instance, Ye et al. conducted a case-control study in Caucasian esophageal-cancer patients and found that the rs14035 polymorphism of RAN was associated with an increased disease risk in a recessive model (*P* = 0.011, OR = 1.99, 95%CI = 1.17 − 3.38) [13]. By contrast, some other researches reported no association between this SNP and the risk of bladder cancer, renal cell carcinoma, and lung cancer [14–16]. The discrepancy might result from the differences in cancer etiology, underlying molecular mechanism, and/or environmental exposure in different population [17]. Therefore, whether the polymorphisms in the RAN gene are cancer-related and how they affect the susceptibility to human cancer remain unclear.

In the present study, we performed a systematic review for the association of RAN SNPs with overall cancer risk. Meanwhile, a meta-analysis was conducted based on available data, aiming at clarifying the association between RAN SNPs and cancer susceptibility. The study may provide novel clues for the identification of miRNA-related genetic biomarkers for the prediction of cancer risk.

## 2. Materials and Methods

### 2.1. Literature Search

Two investigators (Yanke Li and Fuqiang Zhang) independently performed publication search in the database of PubMed and Web of Science up to July 18, 2019, with the following key terms: ‘RAN/Ras-related nuclear', ‘SNP/polymorphism/variant/variation', and ‘cancer/carcinoma/tumor/neoplasm'. All retrieved articles were initially screened for eligible studies according to several standards: (1) case-control study and (2) focused on the association of RAN SNPs with cancer risk. The exclusion criteria contained (1) duplicate records and (2) records irrelevant to RAN SNPs or carcinoma (3) and with no available original data in the publications with authors failing to be contacted with.

### 2.2. Data Extraction

The data was independently extracted by two investigators (Yanke Li and Fuqiang Zhang), and a consensus was reached concerning all items. The information obtained from each article consisted of the first author, publication year, country and ethnicity, sample size, cancer type, SNP genotype distribution in the case and control groups, source of the control group, genotyping method, and adjustment factors. The ethnicity was classified into Asian and Caucasian. The source of controls was divided into hospital-based (HB) and population-based (PB) groups. Both cervical cancer and breast cancer were categorized as “hormone-responsive cancer” in stratified analysis. Bioinformatics function prediction was performed for all involved RAN SNPs using SNPinfo Web Server (https://snpinfo.niehs.nih.gov).

### 2.3. Methodology Quality Assessment

The methodology quality of all enrolled studies after initial screening was independently evaluated by two reviewers (Yanke Li and Fuqiang Zhang) by scoring them based on previous literature [18–20]. Six assessment items were referenced: representativeness of the case group, source of the control group, ascertainment of carcinoma, sample size, quality control of genotyping method, and Hardy-Weinberg equilibrium (HWE). The scores ranged from 0 to 10. The study was judged as eligible for meta-analysis when the quality score was not less than 5.

### 2.4. False-Positive Report Probability

The false-positive report probability (FPRP) was tested to assess the level of statistically significant associations. First, we calculated the statistic power of each association based on the number of observations, and reported ORs and *P* values by using the software NCSS-PASS version 11.0.7 (USA). Then, the FPRP values were figured out followed by the instructions reported in the previous research, and the association with FPRP < 0.5 was considered as a noteworthy finding [21].

### 2.5. Statistical Analysis

The Chi-square test was adopted to evaluate the HWE of SNP genotype frequency distribution in the control group. The association of each SNP with cancer risk was analyzed by calculating the odds ratio (OR) and 95% confidence interval (95% CI). Chi-square-based *Q* statistic test was used to examine the interstudy heterogeneity (statistical significance set as *I*^2^ > 50%). The results in the fixed-effect model were selected when no significant interstudy heterogeneity existed; otherwise, the random-effect model was employed. In addition, the dominant and recessive genetic models were defined as heterozygote+variant homozygote vs. wild homozygote and variant homozygote vs. heterozygote+wild homozygote, respectively. Publication bias was assessed using Begg's rank correlation and Egger's linear regression methods. Sensitivity analysis was performed to evaluate whether the pooled results could be robust to some outlying study. All the above-mentioned analyses were conducted by STATA 11.0 software (STATA Corp., College Station, TX, U.S.A.). *P* < 0.05 was considered to be statistically significant for two-sided tests.

## 3. Results

### 3.1. Characteristics of Eligible Studies

**Figure 1:**
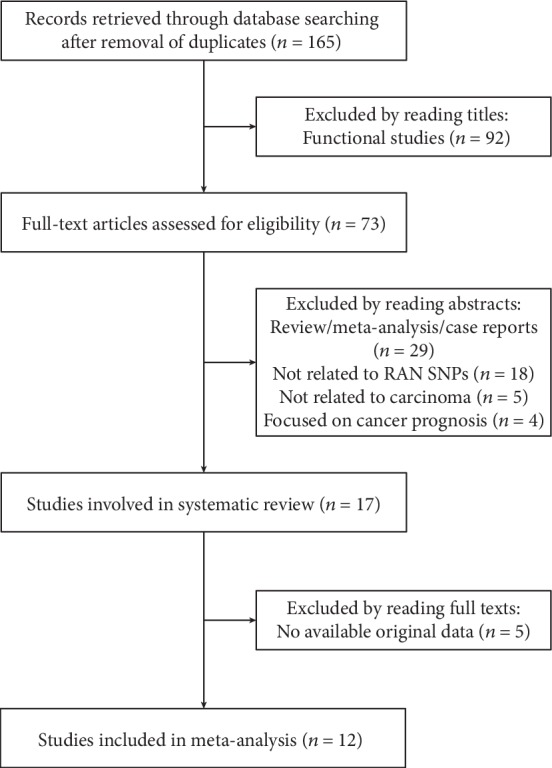
The flow chart of study selection for the meta-analysis.

A total of 165 articles were retrieved through publication search after removing duplicate records. We excluded 148 articles by reading titles and abstracts: 92 were functional studies; 29 were review, meta-analyses, or case reports; 18 were not related to RAN SNPs; 5 were not related to carcinoma; and 4 were focused on cancer prognosis. Therefore, 17 studies were involved in our systematic review. Among them, 5 publications had no available original data, and we failed to contact with their authors. Finally, 12 case-control studies were included in our meta-analysis, consisting of 7662 cases and 9807 controls (Figure 1). The characteristics of eligible studies are presented in Table 1. All of them met the quality assessment.

### 3.2. Characteristics of Involved RAN SNPs

A total of four RAN SNPs were involved in the systematic review, including rs14035 C>T, rs3803012 A>G, rs3809142 C>T, and rs7301722 C>A. Their basic information and function prediction results are shown in [Supplementary-material supplementary-material-1]. The assessment items of SNP biological function mainly comprised of a transcription factor binding site (TFBS), miRNA binding site, RegPotential score, and Conservation score.  Table 2 shows the genotype frequency distribution of all these SNPs. Several records were excluded from quantitative synthesis due to not being in accordance with HWE (*P*_HWE_ < 0.05) or the limited number of some loci. Consequently, two SNPs were included in the final meta-analysis, which were RAN rs14035 and rs3803012 polymorphisms.

### 3.3. Quantitative Data Synthesis of the Association between RAN SNPs and Cancer Risk

**Figure 2:**
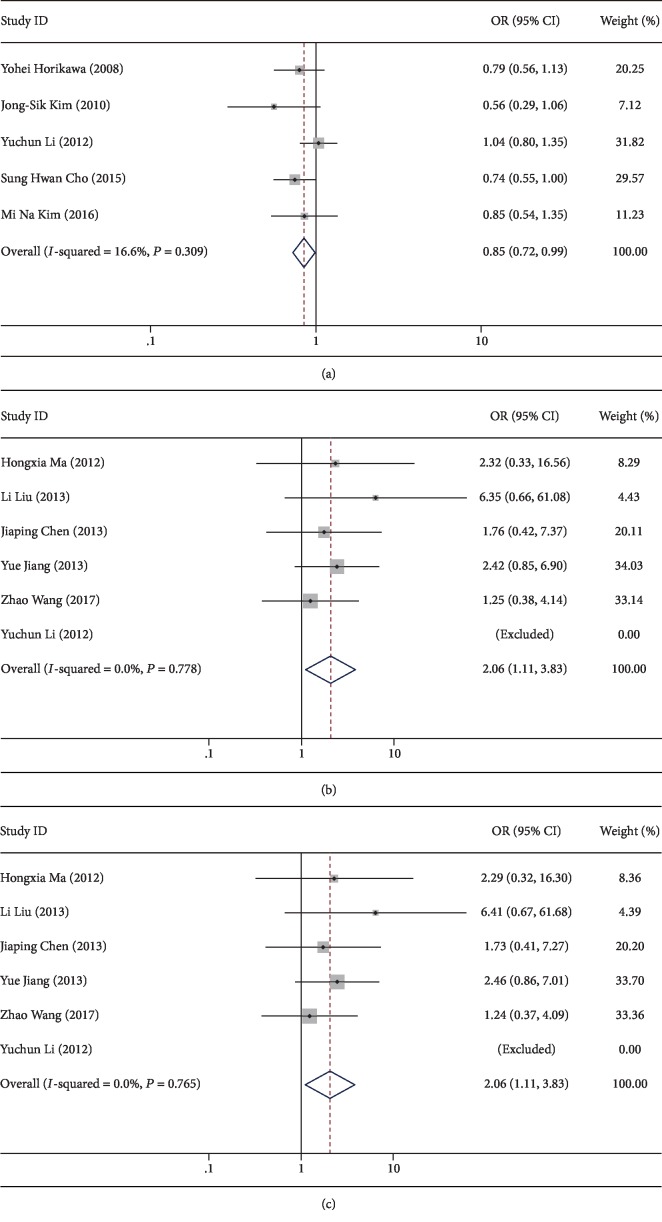
The forest plots of the association between RAN SNPs and cancer risk in overall analysis: (a) the heterozygote model of rs14035 C>T; (b) the variant homozygote model of rs3803012 A>G; (c) the recessive model of rs3803012 A>G.

First, the association of RAN SNPs with overall cancer risk was evaluated by calculating pooled ORs, and both the rs14035 and rs3803012 polymorphisms were found to be associated with cancer risk (Figure 2). The variant CT genotype of rs14035 was linked to a decreased risk when compared with the wild-type CC (*P* = 0.035, OR = 0.85, 95%CI = 0.73 − 0.99). On the contrary, the variant types of rs3803012 conferred to an increased risk (GG vs. AA: *P* = 0.022, OR = 2.06, 95%CI = 1.11 − 3.83; GG vs. AG+AA: *P* = 0.022, OR = 2.06, 95%CI = 1.11 − 3.83, Table 3).

Due to the interstudy heterogeneity, stratified analysis was subsequently performed based on ethnicity, cancer type, and source of controls. Both the rs14035 and rs3803012 polymorphisms also showed significant association with cancer risk in some specific subgroups. In HB population, the variant types of rs14035 could decrease overall cancer risk (CT vs. CC: *P* = 0.011, OR = 0.71, 95%CI = 0.54 − 0.93; CT+TT vs. CC: *P* = 0.009, OR = 0.71, 95%CI = 0.55 − 0.92; and T vs. C: *P* = 0.014, OR = 0.76, 95%CI = 0.61 − 0.95), while no remarkable association was shown in the opposite subgroup. Interestingly, the rs3803012 polymorphism could only elevate the risk in PB population rather than HB population (GG vs. AA: *P* = 0.015, OR = 2.47, 95%CI = 1.19 − 5.13; GG vs. AG+AA: *P* = 0.015, OR = 2.48, 95%CI = 1.19 − 5.14, Table 3).

### 3.4. Sensitivity Analysis

Sensitivity analysis was conducted to estimate the influence of some individual study on pooled results by calculating the ORs before and after exclusion of a single article from meta-analysis in turn. No outlying study was observed to significantly change the pooled ORs after it was removed ([Supplementary-material supplementary-material-1]).

### 3.5. Publication Bias

Furthermore, we evaluated the potential publication bias for all involved studies by using two test methods. No significant publication bias was demonstrated in any genetic model of studied RAN SNPs (Table 4).

### 3.6. FPRP Analysis

To assess the level of positive findings in the meta-analysis, FPRP analysis was performed for all the eight significant associations. According to the published professional guide, studies of rare tumors or small initial studies of common tumors should probably have an FPRP value of 0.5 or above. Given that some estimates of the overall FPRP in the molecular epidemiology literature have been near 0.95, an FPRP value near 0.5 would represent a substantial improvement over the current practice [21, 22]. Now that this is the first report to integrate the association between RNA SNPs and cancer risk with relatively limited studies and sample size involved, we set 0.5 as the FPRP threshold. It was shown that several significant associations of the rs14035 polymorphism (prior probability = 0.25/0.1) could be noteworthy (Table 5).

## 4. Discussion

In this study, a systematic review was conducted for the association of all published SNPs in the RAN gene with the risk of overall cancer. Based on that, a meta-analysis was performed for two highly studied polymorphisms (rs14035 C>T and rs3803012 A>G). The results showed that both the rs14035 and rs3803012 polymorphisms were associated with cancer risk in overall population and some specific subgroups. To our knowledge, it is the first time to make a comprehensive assessment for the research progress in this field and also the first meta-analysis of cancer-related RAN SNPs.

RAN rs14035 SNP, located in the 3′-UTR of the gene, has been widely investigated for its role in carcinogenesis. Although some individual studies reported no significant association between this polymorphism and cancer susceptibility, our meta-analysis suggested that it was linked to a decreased risk of overall cancer. That may result from the limited sample size, ethnic diversity of study population, and complicated environmental factors varied from each study [20]. Additionally, the significance of the rs14035 polymorphism with cancer risk was only presented in the HB group rather than the PB group in stratified analysis. The HB controls were mainly selected from the subjects seeking for physical examination in hospitals, which might have a higher educational level and conferred to the discrepancy. As a locus within the 3′-UTR of a miRNA machinery gene, the rs14035 polymorphism could be responsible for locally altered mRNA secondary structure. For example, 3′-UTR SNPs may lead to different secondary mRNA structures, interfering with RNA-binding proteins, and thus result in distinct allele-dependent differences in mRNA stability [23, 24]. Therefore, the SNPs located in the 3′-UTR of RAN may affect its expression by altering mRNA stability and subsequently participate in cancer genesis and development. In conclusion, the above-mentioned findings demonstrated that RAN rs14035 SNP has the potential to be predictive biomarkers for cancer risk in overall population or some specific subgroup. However, all the hypotheses about related mechanism need to be elucidated by further molecular experiments.

Similar to the rs14035 polymorphism, RAN rs3803012 SNP is also a hotspot 3′-UTR variation with potential biological function. It was suggested to be associated with an increased cancer risk in our study, which was generally consistent with previous research reports. Other than the possible roles, it may exert as a 3′-UTR locus such as rs14035, the A to G changing of rs3803012 might enhance the binding of hsa-miR-199a-3p to the 3′-UTR of RAN. MiR-199a-3p was reported to be a potential candidate for intervention in cancer [25], which was highly expressed in some tumor cells but significantly underexpressed in hepatocellular carcinoma (HCC) and bladder cancer [26–28]. Hence, it could be inferred that RAN rs3803012 G allele might influence the targeting of hsa-miR-199a-3p and lead to reduced expression of RAN mRNA in cancer cells, further affecting a variety of miRNA biological synthesis [17]. Moreover, the SNP function prediction results also indicated that the rs3803012 polymorphism was quite likely to play carcinogenic roles via miRNA-mediated ways. Notably, the association between it and overall cancer risk also showed some differences when stratified by the source of controls. Unlike RAN rs14035, however, the rs3803012 polymorphism contributed to increased disease risk only in the PB group rather than HB group. This phenomenon suggested that the source of controls should be considered as an important influencing factor in the related meta-analysis about this field. Overall, RAN rs3803012 SNP might also become useful biomarkers for predicting cancer risk in general or specific population. Still, further investigations are needed to explore involved molecular mechanism.

Other than the two highly studied polymorphisms mentioned above, another two RAN SNPs (rs3809142 and rs7301722) were also included in our review. However, the original researches referring to them were insufficient in making quantitative synthesis. They were only reported in a case-control study about breast cancer in Chinese women conducted by Jiang et al. The rs7301722 polymorphism was found to be associated with a decreased risk of breast cancer in a codominant model (AA vs. CC: *P* = 0.046, OR = 0.68, 95%CI = 0.47 − 0.99), while no association was observed between the variant types of rs3809142 and disease risk [29]. Both the two SNPs belonged to the promoter region of RAN, and our bioinformatics prediction suggested that they might influence TFBS. As a result, they may affect the expression level of RAN by modulating the transcription initiation site and then change the downstream process. Therefore, RAN rs3809142 and rs7301722 could also have the potential to be functional SNPs as well as biomarkers for cancer risk prediction. Nevertheless, more related studies are needed to be involved in a meta-analysis to clarify the exact association between the two SNPs and overall cancer risk.

It should be acknowledged that our study had some limitations. Foremost, although the meta-analysis has contained a relatively large sample size, the relevant studies remain limited to some extent. It is an emerging field concerning the association of RAN SNPs with cancer risk, and further investigations are request for an updated meta-analysis. In addition, several records without available original data were excluded from final analysis, which might cause publication bias a little.

In summary, we systematically reviewed the association of RAN SNPs with the risk of overall cancer. Furthermore, a meta-analysis was performed using all available data for two highly studied polymorphisms among them (rs14035 and rs3803012). The results showed that both of them were associated with cancer risk in overall population and some specific subgroups, suggesting that they could be potential predictive biomarkers for cancer risk. The study would provide novel clues for the identification of miRNA-related genetic biomarkers applied to predicting cancer susceptibility.

## Figures and Tables

**Table 1:** The characteristics of eligible studies.

Ref. no.	Year	Country	Ethnicity	Sample size		Source of controls	Genotyping method	Adjustment factors	Quality score	Citation
				Case	Control					
1	2008	America	Caucasian	746	746	PB	SNPlex assay	Age, gender, and smoking status	8.5	[14]
2	2008	America	Caucasian	279	278	PB	SNPlex technology	Age, gender, and smoking status	7.5	[15]
3	2010	Korea	Asian	100	100	HB	Sequenome mass spectrometry-based genotyping assay	NM	7	[16]
4	2012	China	Asian	560	560	PB	PCR-RFLP	Gender, age, drinking status, smoking status, and family history of cancer	9.5	[30]
5	2012	China	Asian	397	900	PB	TaqMan allelic discrimination assay	Age, sex, smoking status, and alcohol status	9.5	[31]
6	2013	China	Asian	1300	2688	PB	TaqMan allelic discrimination assay	Age, sex, smoking status, and drinking status	9.5	[17]
7	2013	China	Asian	1486	1549	PB	TaqMan allelic discrimination assay	Age, age at menarche, menopausal status, and parity	9.5	[32]
8	2013	China	Asian	1792	1867	PB	TaqMan OpenArray genotyping system/TaqMan assay	Age, age at menarche, and menopausal status	10	[29]
9	2015	Korea	Asian	408	400	HB	PCR-RFLP	Age, gender, hypertension, and diabetes mellitus	5	[33]
10	2015	Poland	Caucasian	135	170	HB	TaqMan SNP genotyping assay	NM	5.5	[34]
11	2016	Korea	Asian	147	229	PB	PCR-RFLP	Age, gender, hypertension, diabetes mellitus, drinking status, and smoking	5.5	[35]
12	2017	China	Asian	312	320	HB	TaqMan SNP genotyping assay	Age, gender, smoking status, alcohol intake, hepatitis B/C virus infection, and fatty liver/nonalcoholic hepatitis status	7	[36]

Note: PB, population-based; HB, hospital-based; PCR-RFLP, in reaction-restriction fragment length polymorphism; NM, not mentioned.

**Table 2:** The genotype frequency distribution of RAN SNPs in enrolled studies.

Ref. no.	Year	Cancer type	SNP^a^	Sample size			Case			Control		*P*_HWE_	Included in meta-analysis
				Case	Control	Wild homozygote	Heterozygote	Variant homozygote	Wild homozygote	Heterozygote	Variant homozygote		
1	2008	Bladder cancer	rs14035 C>T	746	746	348	329	58	318	351	63	*0.013*	No^b^
2	2008	RCC	rs14035 C>T	279	278	143	110	23	129	125	24	0.415	Yes
3	2010	Lung cancer	rs14035 C>T	100	100	65	23	5	52	33	5	0.937	Yes
4	2012	HCC	rs14035 C>T	560	560	376	160	24	390	160	10	0.162	Yes
			rs3803012 A>G	560	560	508	52	0	512	48	0	0.289	Yes
5	2012	HNC	rs3803012 A>G	397	900	344	45	2	799	91	2	0.725	Yes
6	2013	HCC	rs3803012 A>G	1300	2688	1158	95	3	2450	227	1	0.066	Yes
7	2013	Cervical cancer	rs3803012 A>G	1486	1549	1325	141	5	1397	129	3	0.990	Yes
8	2013	Breast cancer	rs3809142 C>T	1792	1867	602	232	28	615	239	32	0.149	No^c^
			rs3803012 A>G	1792	1867	766	92	12	772	107	5	0.539	Yes
			rs7301722 C>A	1792	1867	881	733	131	966	716	174	*0.015*	No^b/c^
9	2015	CRC	rs14035 C>T	408	400	267	128	13	233	150	17	0.240	Yes
10	2015	Larynx cancer	rs14035 C>T	135	170	73	32	5	67	93	10	*0.002*	No^b^
11	2016	HCC	rs14035 C>T	147	229	98	42	7	137	69	3	0.080	Yes
12	2017	HCC	rs3803012 A>G	312	320	250	56	6	260	55	5	0.298	Yes

Note: RCC, renal cell carcinoma; HCC, hepatocellular carcinoma; HNC, head and neck cancer; CRC, colorectal cancer; *P*_HWE_, Hardy-Weinberg equilibrium in the control group. ^a^Ancestral alleles were referenced in NCBI database. ^b^Excluded due to not being in accordance with HWE. ^c^Excluded due to the limited number of this locus. The results are in italics if *P* < 0.05.

**Table 3:** The meta-analysis of the association between RAN SNPs and cancer risk.

SNP	*N*	Heterozygote vs. wild homozygote			Variant homozygote vs. wild homozygote			Dominant model			Recessive model			Allelic model		
		*P*	OR (95% CI)	*I* ^2^ (%)	*P*	OR (95% CI)	*I* ^2^ (%)	*P*	OR (95% CI)	*I* ^2^ (%)	*P*	OR (95% CI)	*I* ^2^ (%)	*P*	OR (95% CI)	*I* ^2^ (%)
rs14035 C>T	5	*0.035*	0.85 (0.73-0.99)	16.6	0.520^a^	1.22 (0.67-2.24)	57.7	0.102	0.88 (0.76-1.03)	43.9	0.338^a^	1.31 (0.75-2.27)	50.6	0.452^a^	0.93 (0.76-1.13)	55.9
Ethnicity																
Asian	4	0.086	0.86 (0.72-1.02)	35.3	0.425^a^	1.39 (0.62-3.15)	63.5	0.311^a^	0.87 (0.66-1.14)	55.6	0.293^a^	1.49 (0.71-3.15)	57.4	0.629^a^	0.94 (0.72-1.22)	65.1
Caucasian	1	0.196	0.79 (0.56-1.13)	NA	0.645	0.87 (0.47-1.61)	NA	0.203	0.81 (0.58-1.12)	NA	0.899	0.96 (0.53-1.75)	NA	0.299	0.87 (0.67-1.13)	NA
Source of controls																
HB	2	*0.011*	0.71 (0.54-0.93)	0.0	0.273	0.70 (0.37-1.33)	0.0	*0.009*	0.71 (0.55-0.92)	0.0	0.472	0.79 (0.42-1.50)	0.0	*0.014*	0.76 (0.61-0.95)	0.0
PB	3	0.431	0.93 (0.77-1.12)	0.0	0.216^a^	1.72 (0.73-4.05)	66.7	0.892	0.99 (0.82-1.19)	18.7	0.153^a^	1.77 (0.81-3.89)	61.7	0.532	1.05 (0.90-1.22)	38.9
rs3803012 A > G	6	0.913	1.01 (0.89-1.14)	0.0	*0.022*	2.06 (1.11-3.83)	0.0	0.587	1.04 (0.91-1.17)	0.0	*0.022*	2.06 (1.11-3.83)	0.0	0.324	1.06 (0.94-1.20)	0.0
Cancer type																
HCC	3	0.676	0.96 (0.80-1.16)	0.0	0.240	1.85 (0.66-5.16)	35.6	0.827	0.98 (0.81-1.18)	0.0	0.245	1.84 (0.66-5.12)	37.2	0.999	1.00 (0.84-1.19)	0.0
Hormone-responsive cancer	2	0.940^a^	1.01 (0.77-1.34)	51.9	0.071	2.17 (0.94-5.05)	0.0	0.533	1.06 (0.88-1.28)	23.5	0.069	2.19 (0.94-5.08)	0.0	0.312	1.10 (0.92-1.31)	0.0
HNC	1	0.474	1.15 (0.79-1.68)	NA	0.400	2.32 (0.33-16.56)	NA	0.400	1.17 (0.81-1.70)	NA	0.409	2.29 (0.32-16.30)	NA	0.341	1.19 (0.83-1.70)	NA
Source of controls																
HB	1	0.785	1.06 (0.70-1.60)	NA	0.717	1.25 (0.38-4.14)	NA	0.721	1.08 (0.72-1.60)	NA	0.729	1.24 (0.37-4.09)	NA	0.668	1.08 (0.76-1.55)	NA
PB	5	0.979	1.00 (0.88-1.14)	0.0	*0.015*	2.47 (1.19-5.13)	0.0	0.649	1.03 (0.90-1.18)	0.0	*0.015*	2.48 (1.19-5.14)	0.0	0.371	1.06 (0.93-1.20)	0.0

Note: ^a^*P* was calculated by the random model; OR, odds ratio; CI, confidence interval; NA, not available. The results are in italics if *P* < 0.05.

**Table 4:** Begg's and Egger's tests for publication bias.

Comparison type	Begg's test		Egger's test	
	*Z* value	*P* value	*t* value	*P* value
rs14035 C>T				
Heterozygote vs. wild homozygote	0.73	0.462	-1.55	0.220
Variant homozygote vs. wild homozygote	0.73	0.462	0.61	0.587
Dominant model	0.24	0.806	-1.09	0.354
Recessive model	0.73	0.462	0.74	0.513
Allelic model	0.24	0.806	-0.63	0.572
rs3803012 A>G				
Heterozygote vs. wild homozygote	0.38	0.707	0.66	0.543
Variant homozygote vs. wild homozygote	0.73	0.462	1.12	0.345
Dominant model	0.38	0.707	0.72	0.513
Recessive model	0.73	0.462	1.06	0.367
Allelic model	0.00	1.000	0.68	0.532

Note: The results are in italics if *P* < 0.1.

**Table 5:** FPRP values for the association between RAN SNPs and cancer risk.

Genotype	OR (95% CI)	*P*	Statistical power^a^	Prior probability^b^				
				0.25	0.1	0.01	0.001	0.0001
rs14035 C>T								
CT vs. CC (overall)	0.85 (0.73-0.99)	0.035	0.226	*0.453*	0.633	0.940	0.994	0.999
CT vs. CC (HB)	0.71 (0.54-0.93)	0.011	0.227	*0.205*	*0.350*	0.830	0.980	0.998
CT+TT vs. CC (HB)	0.71 (0.55-0.92)	0.009	0.233	*0.171*	*0.301*	0.796	0.975	0.997
T vs. C (HB)	0.76 (0.61-0.95)	0.014	0.300	*0.199*	*0.341*	0.825	0.979	0.998
rs3803012 A>G								
GG vs. AA (overall)	2.06 (1.11-3.83)	0.022	<0.001	0.992	0.996	1.000	1.000	1.000
GG vs. AG+AA (overall)	2.06 (1.11-3.83)	0.022	<0.001	0.992	0.996	1.000	1.000	1.000
GG vs. AA (PB)	2.47 (1.19-5.13)	0.015	<0.001	0.988	0.994	0.999	1.000	1.000
GG vs. AG+AA (PB)	2.48 (1.19-5.14)	0.015	<0.001	0.988	0.994	0.999	1.000	1.000

Note: FPRP, false-positive report probability; OR, odds ratio; CI, confidence interval. ^a^The statistical power is calculated using the number of observations and ORs and *P* values. ^b^The results are in italics if FPRP < 0.5.

## Abbreviations

miRNA: MicroRNA
SNP: Single nucleotide polymorphism
HB: Hospital-based
PB: Population-based
HWE: Hardy-Weinberg equilibrium
OR: Odds ratio
CI: Confidence interval
TFBS: Transcription factor binding site
HCC: Hepatocellular carcinoma.

## Supplementary Materials

Supplementary Materials Table S1: the function prediction of RAN SNPs. Table S2: The ORs (95% CIs) of sensitivity analysis.
