# Supplementary material for: A Systematic Review and Meta-Analysis for the Association of Gene Polymorphisms in RAN with Cancer Risk
Source: Dis Markers. 2020 Jan 16;2020:9026707. doi: 10.1155/2020/9026707 (PMC6985935; doi:10.1155/2020/9026707)
Supplement: Supplementary Materials — Table S1: the function prediction of RAN SNPs. Table S2: The ORs (95% CIs) of sensitivity analysis. [file 9026707.f1.docx]

| Table S1. The function prediction of RAN SNPs | | | | |
| --- | --- | --- | --- | --- |
| SNP function item | rs14035 | rs3803012 | rs3809142 | rs7301722 |
| Allele | C/T | A/G | C/T | C/A |
| Position | 3'-UTR | 3'-UTR | Promoter | Promoter |
| nsSNP | -- | -- | -- | -- |
| Splicing (site) | -- | -- | -- | -- |
| Splicing (abolish domain) | -- | -- | -- | -- |
| Splicing (ESE or ESS) | -- | -- | -- | -- |
| Stop Codon | -- | -- | -- | -- |
| Polyphen | -- | -- | -- | -- |
| SNPs3D (svm profile) | -- | -- | -- | -- |
| SNPs3D (svm structure) | -- | -- | -- | -- |
| TFBS | -- | -- | Y | Y |
| miRNA (miRanda) | Y | Y | -- | -- |
| miRNA (Sanger) | -- | Y | -- | -- |
| RegPotential | 0.0 | 0.315581 | NA | NA |
| Conservation | 0.000 | 1.000 | 0.000 | 0.000 |
| Note: nsSNP, nonsynonymous SNP; ESE, exon splicing enhancer; ESS, exon splicing silencer; TFBS, transcription factor binding site; NA,not available. | | | | |
|  |  |  |  |  |

| Table S2. The ORs (95%CIs) of sensitivity analysis | | | | | |
| --- | --- | --- | --- | --- | --- |
| First author (Year) | Heterozygote vs. Wild homozygote | Variant homozygote vs. Wild homozygote | Dominant model | Recessive model | Allelic model |
|  |  |  |  |  |  |
|  | OR(95%CI) | OR(95%CI) | OR(95%CI) | OR(95%CI) | OR(95%CI) |
| **rs14035 C>T** |  |  |  |  |  |
| Overall | 0.84(0.70-1.00) | 1.22(0.67-2.24) | 0.86(0.70-1.06) | 1.31(0.75-2.27) | 0.93(0.76-1.13) |
| Yohei Horikawa (2008) | 0.84(0.66-1.06) | 1.39(0.62-3.15) | 0.87(0.66-1.14) | 1.49(0.71-3.15) | 0.94(0.72-1.22) |
| Jong-Sik Kim (2010) | 0.87(0.74-1.02) | 1.32(0.65-2.69) | 0.90(0.73-1.11) | 1.39(0.73-2.67) | 0.96(0.78-1.19) |
| Yuchun Li (2012) | 0.76(0.62-0.92) | 0.93(0.55-1.57) | 0.78(0.64-0.93) | 1.03(0.63-1.68) | 0.84(0.72-0.98) |
| Sung Hwan Cho (2015) | 0.87(0.70-1.08) | 1.47(0.73-2.97) | 0.91(0.71-1.16) | 1.57(0.83-2.94) | 0.98(0.79-1.22) |
| Mi Na Kim (2016) | 0.82(0.66-1.03) | 1.06(0.57-1.96) | 0.84(0.65-1.08) | 1.15(0.67-1.99) | 0.90(0.71-1.14) |
| **rs3803012 A>G** |  |  |  |  |  |
| Overall | 1.01(0.89-1.14) | 2.04(1.09-3.81) | 1.04(0.91-1.17) | 2.04(1.09-3.81) | 1.06(0.94-1.20) |
| Yuchun Li (2012) | 1.00(0.87-1.14) | 2.04(1.09-3.81) | 1.03(0.90-1.17) | 2.04(1.09-3.81) | 1.06(0.94-1.20) |
| Hongxia Ma (2012) | 0.99(0.87-1.13) | 2.01(1.04-3.89) | 1.02(0.89-1.16) | 2.01(1.04-3.89) | 1.05(0.92-1.19) |
| Li Liu (2013) | 1.05(0.91-1.22) | 1.85(0.97-3.56) | 1.08(0.94-1.25) | 1.85(0.96-3.55) | 1.11(0.96-1.27) |
| Jiaping Chen (2013) | 0.96(0.83-1.12) | 2.11(1.05-4.23) | 1.00(0.86-1.15) | 2.11(1.05-4.24) | 1.03(0.90-1.18) |
| Yue Jiang (2013) | 1.04(0.91-1.20) | 1.85(0.85-4.04) | 1.06(0.92-1.22) | 1.83(0.84-4.00) | 1.08(0.94-1.23) |
| Zhao Wang (2017) | 1.00(0.88-1.15) | 2.45(1.17-5.10) | 1.03(0.90-1.18) | 2.46(1.18-5.12) | 1.06(0.93-1.20) |
| Note: OR, odds ratio; CI, confidence interval. | | | | | |
